# Diversity matters in scientific publishing

**DOI:** 10.1186/1742-4690-9-109

**Published:** 2012-12-19

**Authors:** Kuan-Teh Jeang

**Affiliations:** 1 The National Institutes of Health, Bethesda, MD, USA

## Abstract

The importance of geographic diversity in publishing is emphasized in this editorial.

## 

Earlier this year, I read about a remarkable change in the United States population. For the first time in United States history, the majority (50.4%) of children 1 year and younger are from minority groups (e.g. Hispanic American, African American, Asian American etc.…). In fact, the face of America is changing not just amongst her young but in its totality. Population projections show by the year 2043, the United States population will exceed 400 million; and minority groups will compose a majority of this number. Already four states in the United States (Texas, California, Hawaii, and New Mexico) are “majority-minority” states where the combined population of various “minorities” exceeds the erstwhile “white majority” number.

The face of global science is also changing. I have previously written about the rise of bioscience in the East [1]. A Royal Society report (http://royalsociety.org/policy/projects/knowledge-networks-nations/report/), surveying the knowledge, networks and nations landscape in 2011, confirmed this notion and projected that by 2013 the total number of scientific publications from China published in English will overtake the counterpart number from the United States. Anecdotally, in recent years each of us has seen a steady increase in the number of papers published in our leading biomedical journals by Chinese and Asian authors. Surprisingly, in the face of these trends, a longstanding publishing inherency appears to hold sway. Thus, last year in a newsletter to its editors, the largest publisher of biomedical literature Elsevier reported that “while the spread of countries represented on the editorial boards of Elsevier journals is ‘reasonable’, countries such as India and China are under-represented in comparison with their share of published articles. Interestingly, the percentage of Elsevier editors from China is 3.3% while nearly 13% of published articles originate there. Some countries are also significantly over-represented, for example, 40% of our editors come from the US while only 18% of published articles originate there…” Needless to say, this imbalance should be remedied.

In this regard, how is the editorial diversity at *Retrovirology*? Amongst the 8 *Retrovirology *editors, 6 are from countries outside of North America (a 75% non-North American representation). In *Retrovirology*’*s *60 member editorial board, 32 individuals hail from outside of North America (a 53% majority). We believe that diversity is important because we hold firm the idea that intelligence and ambition are distributed equally around the globe [2]. One area that we need to do better is to publish more papers from authors outside of the traditional bastions of North American and West European science. In examining all papers published last year, I found that only 10 published *Retrovirology *papers came from authors based outside of North America and Western Europe [3-12]; and these papers were submitted from either Australia or Japan. Although peer-reviewed quality remains the criterion for publication, our goal is to strongly encourage and work hard in attracting to *Retrovirology *more manuscripts from diverse areas of the world. Diversity in science and scientific publishing matters.
